# Effects of different aperture-sized type I collagen/silk fibroin scaffolds on the proliferation and differentiation of human dental pulp cells

**DOI:** 10.1093/rb/rbab028

**Published:** 2021-06-25

**Authors:** Shihui Jiang, Zhaoxia Yu, Lanrui Zhang, Guanhua Wang, Xiaohua Dai, Xiaoli Lian, Yan Yan, Linpu Zhang, Yue Wang, Ruixin Li, Huiru Zou

**Affiliations:** Tianjin Key Laboratory of Oral and Maxillofacial Function Reconstruction, Tianjin Stomatological Hospital, The Affiliated Stomatological Hospital of Nankai University, No. 75 Dagu Road, Heping District, Tianjin 300041, China; Tianjin Key Laboratory of Oral and Maxillofacial Function Reconstruction, Tianjin Stomatological Hospital, The Affiliated Stomatological Hospital of Nankai University, No. 75 Dagu Road, Heping District, Tianjin 300041, China; Tianjin Key Laboratory of Oral and Maxillofacial Function Reconstruction, Tianjin Stomatological Hospital, The Affiliated Stomatological Hospital of Nankai University, No. 75 Dagu Road, Heping District, Tianjin 300041, China; Tianjin Key Laboratory of Oral and Maxillofacial Function Reconstruction, Tianjin Stomatological Hospital, The Affiliated Stomatological Hospital of Nankai University, No. 75 Dagu Road, Heping District, Tianjin 300041, China; Tianjin Key Laboratory of Oral and Maxillofacial Function Reconstruction, Tianjin Stomatological Hospital, The Affiliated Stomatological Hospital of Nankai University, No. 75 Dagu Road, Heping District, Tianjin 300041, China; Tianjin Key Laboratory of Oral and Maxillofacial Function Reconstruction, Tianjin Stomatological Hospital, The Affiliated Stomatological Hospital of Nankai University, No. 75 Dagu Road, Heping District, Tianjin 300041, China; Tianjin Key Laboratory of Oral and Maxillofacial Function Reconstruction, Tianjin Stomatological Hospital, The Affiliated Stomatological Hospital of Nankai University, No. 75 Dagu Road, Heping District, Tianjin 300041, China; Tianjin Key Laboratory of Oral and Maxillofacial Function Reconstruction, Tianjin Stomatological Hospital, The Affiliated Stomatological Hospital of Nankai University, No. 75 Dagu Road, Heping District, Tianjin 300041, China; Tianjin Key Laboratory of Oral and Maxillofacial Function Reconstruction, Tianjin Stomatological Hospital, The Affiliated Stomatological Hospital of Nankai University, No. 75 Dagu Road, Heping District, Tianjin 300041, China; School of Medicine, Nankai University, No. 94 Weijin Road, Nankai District, Tianjin 300071, China; Tianjin Key Laboratory of Oral and Maxillofacial Function Reconstruction, Tianjin Stomatological Hospital, The Affiliated Stomatological Hospital of Nankai University, No. 75 Dagu Road, Heping District, Tianjin 300041, China; Tianjin Key Laboratory of Oral and Maxillofacial Function Reconstruction, Tianjin Stomatological Hospital, The Affiliated Stomatological Hospital of Nankai University, No. 75 Dagu Road, Heping District, Tianjin 300041, China; School of Medicine, Nankai University, No. 94 Weijin Road, Nankai District, Tianjin 300071, China; State Key Laboratory of Medicinal Chemical Biology, Nankai University, No. 94 Weijin Road, Nankai District, Tianjin 300071, China

**Keywords:** tissue engineering, dentine-pulp complex regeneration, collagen, silk fibroin, odontogenic differentiation

## Abstract

This study aimed at evaluate the effects of different aperture-sized type I collagen/silk fibroin (CSF) scaffolds on the proliferation and differentiation of human dental pulp cells (HDPCs). The CSF scaffolds were designed with 3D mapping software Solidworks. Three different aperture-sized scaffolds (CSF1–CSF3) were prepared by low-temperature deposition 3D printing technology. The morphology was observed by scanning electron microscope (SEM) and optical coherence tomography. The porosity, hydrophilicity and mechanical capacity of the scaffold were detected, respectively. HDPCs (third passage, 1 × 10^5^ cells) were seeded into each scaffold and investigated by SEM, CCK-8, alkaline phosphatase (ALP) activity and HE staining. The CSF scaffolds had porous structures with macropores and micropores. The macropore size of CSF1 to CSF3 was 421 ± 27 μm, 579 ± 36 μm and 707 ± 43 μm, respectively. The porosity was 69.8 ± 2.2%, 80.1 ± 2.8% and 86.5 ± 3.3%, respectively. All these scaffolds enhanced the adhesion and proliferation of HDPCs. The ALP activity in the CSF1 group was higher than that in the CSF3 groups (*P *<* *0.01). HE staining showed HDPCs grew in multilayer within the scaffolds. CSF scaffolds significantly improved the adhesion and ALP activity of HDPCs. CSF scaffolds were promising candidates in dentine-pulp complex regeneration.

## Introduction

When dental tissue is damaged due to trauma or infection, various treatments will be chosen clinically according to different situations, such as direct pulp capping, indirect pulp capping, vital pulpotomy, root canal therapy, etc. The final goal is to restore the function and integrity of the tooth structure, while maintaining the natural appearance. Although the above treatments can restore the damaged tooth to a certain extent, they cannot replace natural healthy intact dental tissues [1]. With the continuous development of bioactive materials and tissue engineering technology, dentine–pulp complex regeneration is expected to be a new treatment option, which may replace the current treatments to repair tooth defects and restore tooth function [2].

Scaffolds prepared by 3D printing technology possess highly precised three-dimensional structures. They are expected to provide suitable microenvironments for cell adhesion and growth, facilitate signal transmission between cells and enhance the functional activity of cells. Undoubtedly, an ideal scaffold material for dentine–pulp complex regeneration needs to have good biocompatibility, controllable biodegradability, suitable mechanical property and porosity, good surface adhesion property, etc [3]. As a main component of extracellular matrix, type I collagen plays important roles in bone, cartilage, skin, vessel, nerve and ligament regeneration due to its superior biocompatibility, low immunogenicity and suitable biodegradation properties [4–6]. Several studies have shown that type I collagen is one of the most ideal scaffold materials for dental pulp regeneration [7, 8]. It can significantly enhance the growth of human dental pulp cells (HDPCs) and increase the odontogenic related gene expression. However, due to its poor mechanical properties, poor thermal stability and fast biodegradability, the physical and structural properties of the collagen-based scaffold need to be improved. Silk fibroin, a natural material spun by silkworms, has attracted the attention of many researchers in recent years. It has advantages of easy processing, excellent mechanical properties, outstanding biocompatibility, suitable permeability. Meanwhile, silk fibroin is less immunogenic than other reported biomaterials [9, 10]. Previous study reported that HDPCs cultured in pure silk fibroin scaffold showed angiogenic ability, which was important for dental pulp regeneration [11]. Pure silk scaffold enhanced the attachment, proliferation and angiogenic differentiation of HDPCs *in vitro*. But, *in vivo* experiments identified that the amount of cells and number of blood vessels in pure silk scaffold group was insufficient compared to basic fibroblast growth factor (bFGF) incorporated silk scaffold group. In addition, no dentine-like tissue and mineral deposition were generated [12]. These findings suggested that single collagen or silk scaffold could not meet the requirement of dental pulp regeneration.

A type of 3D collagen/silk fibroin scaffold with cavity was fabricated recently, simulating the anatomy of normal spinal cord. Results showed that this scaffold mimicked 3D microenvironment and promoted the repair of injured spinal cord [13]. Our group constructed a type II collagen/silk fibroin scaffold with suitable morphology, physical stability and biological functionality to promote chondrocytes proliferation [14]. Consistent results were also obtained by other teams confirmed that such materials had a favorable effect on articular cartilage repair [15, 16]. However, no attempts had been made on the application of such materials on dentine–pulp complex regeneration. Collagen/silk fibroin scaffold might overcome single material’s shortcomings and contribute to the development of dental tissue engineering.

**Figure 1.**
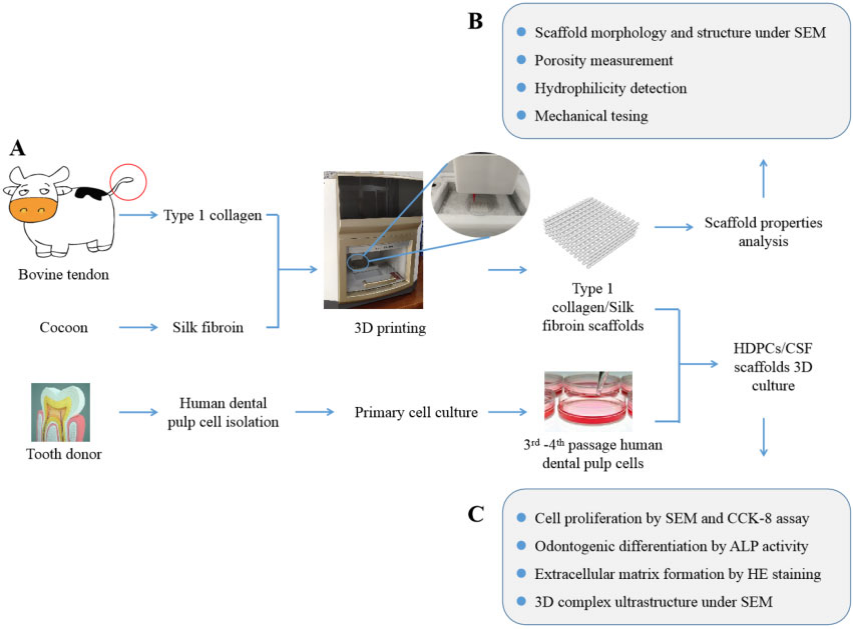
Schematics of the whole study, including the fabrication of the type I collagen/silk fibroin scaffold and human dental pulp cell-scaffold culture.

Previous studies also showed that pore architectures of the scaffolds influence the cell migration and infiltration. The osteochondral tissue regeneration was obviously faster in the radially aligned scaffold group than in the axially aligned pore or random pore groups [15]. Meanwhile, different pore size had different effects on cell proliferation and cell migration [17, 18]. Therefore, the aim of this study is to fabricate the type I collagen/silk fibroin (CSF) scaffolds with different aperture sizes by low-temperature deposition 3D printing technology, and to investigate the properties of CSF scaffolds and the effects of these scaffolds on the proliferation and differentiation of HDPCs, in order to explore the possible application of CSF scaffolds in dentine–pulp complex regeneration. Schematics of the whole study were shown in Fig. 1.

## Materials and methods

### Preparation of CSF scaffold

#### Preparation of type I collagen

Type I collagen was extracted from bovine tendon by the acid swelling–pepsin digestion method. Briefly, the fresh bovine tendon fat was removed. Then, the residues were pulverized and soaked in 0.05 mol/l Tris buffer for 24 h. The precipitate was collected. Then the acetic acid solution containing pepsin was added, and the supernatant was collected. After that, 3.5 mol/l NaCl solution was added for salting out. Deionized water was used for the dialysis at 4°C for 5 days. In order to calculate the concentration of the collagen solution, culture dishes were used. The dry and clean culture dish was weighed as M_0_. The appropriate amount of collagen solution was added to the culture dish, and the weight was recorded as M_1_. Then the culture dish was placed in a 60°C constant temperature drying oven until the collagen was fully dried. The weight was recorded as M_2_. The concentration of collagen solution was calculated according to the formula: (M_2_ − M_0_)/(M_1_ − M_0_) × 100%, the above experiment was repeated three times and the mean value was taken.

#### Preparation of silk fibroin

Silk fibroin was produced using cocoons following the method described below. In brief, 100 g silk was boiled in a 0.5% anhydrous Na_2_CO_3_ solution for 30 min for degumming, and repeated three times, then the silk was washed with deionized water and dried in a 60°C constant temperature drying oven. Then, the dried silk was placed in a three-necked flask containing a CaCl_2_·CH_3_CH_2_OH·H_2_O ternary solution with a molar ratio of 1:2:8 at 80°C, mechanically stirred for 2 h, then cooled, and placed in a dialysis bag, and dialyzed with tap water for 48 h, then dialyzed with deionized water. The deionized water was replaced every 8 h. After dialysis for 3 days, the dialysis bag containing silk fibroin was concentrated in a 40% polyethylene glycol solution for 12 h and stored at 4°C. The same concentration determination method was used for silk fibroin concentration measurement as collagen.

#### Fabrication of CSF scaffolds

A three-dimensional scaffold printing model of standard tessellation language (STL) format was designed using the design software Solidworks 2010 (SolidWorks Corporation, Waltham, MA, USA). The model was imported into the computer control software of 3D printer (OrganP 1800, Organprinter, China). Printing parameters were as follows: normal feed speed 10 mm/s, ordinary extrusion speed 2 mm/s, first layer print height correction 0.7 mm, print layer thickness 0.32 mm, print layer 7 layers, print needle diameter 260 μm, bottom plate molding temperature −20°C. The collagen and silk fibroin with the mass ratio of 1:1 were fully mixed in a three-necked bottle and mixed in the charging basket of printer. Three different aperture-sized scaffolds were printed according to the number of lines per scaffold (25, 22 and 20 lines, respectively). The scaffolds were lyophilized in a vacuum freeze dryer overnight, then treated with anhydrous ethanol and 0.01% NaOH solution for 24 h, then repeatedly rinsed with deionized water, and sterilized by exposure to 20 kGy Co^60^ and immersed in sterile saline for 30 min before use.

### Morphology and structure observation

After coated with gold using a JEOL JFC-110E Ion Sputter, the scaffolds were observed under a scanning electron microscope (SU8100, HITACHI, Japan). Optical coherence tomography (OCTMI, OCT Medical Imaging Inc., Irvine, USA) was also used to observe the morphology of the scaffold. Three-dimensional reconstructions of the samples were made during post processing using Image J (National Institutes of Health, Bethesda, MD, USA).

### Porosity

The porosity of CSF scaffolds was determined by a modified liquid replacement method. The anhydrous ethanol with a volume of V_1_ was added to the measuring cylinder. The CSF scaffolds were placed in the cylinder and immersed in ethanol for 5 min. The cylinder was placed in the vacuum pump until no bubbles emitted. The ethanol volume was recorded as V_2_. Then the scaffolds were taken out gently. The remaining ethanol volume was recorded as V_3._ The porosity of the scaffold was calculated according to the equation: *p* (%)= (*V*_1_ − *V*_3_)/(*V*_2_ − *V*_3_) × 100%. Three samples were taken from each group and the mean value was calculated.

### Hydrophilicity

The completely dried scaffolds were weighed as W_1_. Then the scaffolds were immersed in distilled water at room temperature and taken out after 24 h. The filter paper was used to dry the water on the surface of the scaffolds. Then the scaffolds were weighed as W_2_. The hydrophilicity was calculated by the following equation: *h* (%) = (*W*_2_ − *W*_1_)/*W*_1_ × 100%. Three samples were taken from each group and the mean value was calculated.

### Mechanical testing

The mechanical properties of the scaffolds were examined using a universal testing machine (Instron5865, USA). Before the mechanical experiment, the scaffolds were immersed in the PBS solution for 24 h. Tests were carried out using a 0.01 N preload at a velocity of 2 mm/min. The maximum compressive strain was 10%. Three samples were measured in each group. The stress–strain curve was plotted. Then the compressive modulus was defined as the slope of a linear fit to the stress–strain curve over 1–5% strain.

### HDPCs–CSF scaffolds culture

Human dental pulp cells (HDPCs) were isolated from human dental pulp tissue using outgrowth/explants culture method. Briefly, the sound premolar or the third molar teeth freshly extracted due to orthodontics reasons were collected at the surgical clinic of Tianjin Stomatological Hospital with the informed consent from the patients. The patients were 16–28 years old. The teeth were soaked in alpha minimum essential medium supplemented with 100 U/ml penicillin and streptomycin. After sterilized for 20 min at 4°C, the teeth were cracked open with a bone hammer. The extracted pulp tissue was immersed in a PBS solution containing 100 U/ml penicillin and streptomycin for 1 min, then placed in 20% FBS α-MEM medium, and cut into 1 mm × 1 mm × 1 mm pieces by ophthalmic scissors, and then transferred into a culture flask containing 3 ml 20% FBS α-MEM medium for inversion culture. After 4 h, the culture flask was slowly inverted. When the cells reached 90% confluence, cell passage was carried out with 0.25% trypsin. The 3^rd^ passage HDPCs (1 × 10^5^ cells) were seeded into each scaffold, and the cell-scaffold complex was cultured in a 37°C, 5% CO_2_ incubator. After 24 h, each cell-scaffold complex was transferred to a new 24-well plate and cultured in 500 μl of 15% FBS αMEM medium. The medium was renewed every 3 days.

### SEM observation on the adhesion and proliferation of HDPCs on the scaffolds

The scaffolds were taken out 1 day and 7 days after incubation. The scaffolds were rinsed with PBS three times and then fixed with 2.5% glutaraldehyde overnight at 4°C. SEM was used to observe the adhesion and proliferation of HDPCs on the scaffolds.

### Quantitative analysis of cell proliferation

After 1, 3, 5 and 7 days culture, 50 μl CCK-8 solution was added to each well of the 24-well plate. The culture plates were incubated in the incubator for 4 h, then 100 μl liquid from each well was added to the 96-well plate, and measured spectrophotometrically at 492 nm wavelength.

### Alkaline phosphatase activity

After 7 and 14 days culture, the cell-scaffold complex was fully cut into pieces and immersed in lysis solution. After achieving the complete lysis, the complex was centrifuged at 12 000 rpm for 5 min at 4°C. Using the ALP assay kit, incubated for 30 min at 37°C, the absorbance of p-nitrophenyl-phosphate at 405 nm was measured with a microplate reader. ALP activity was expressed as substrate hydrolyzed mol p-nitropheno/min.

### Histological observation

After 14 days culture, the cell-scaffold complexes were taken out from the incubator and rinsed with PBS. The excess liquid on the scaffolds were blotted with filter paper. The samples were fixed with 4% paraformaldehyde solution at room temperature overnight. After embedded with paraffin, the scaffolds were continuously sliced at a thickness of 5 μm, and then processed for HE staining observation.

### Statistical analysis

The statistical analyses were performed using SPSS 20.0 software. Data were expressed as mean ± standard deviation (Mean ± SD). One-way analysis of variance (ANOVA) was used to compare the results. A *P *<* *0.05 was considered to be statistically significant.

## Results

### Morphology of the CSF scaffolds

**Figure 2.**
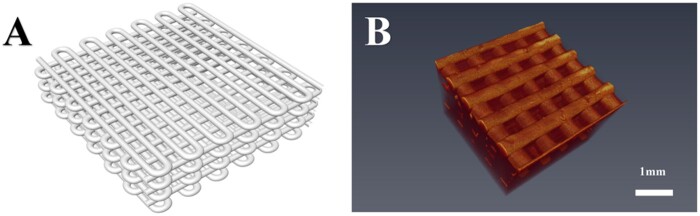
The type I collagen/silk fibroin (CSF) scaffold designed in this study. **A**: The model of STL format was designed using the design software Solidworks 2010; **B**: Optical coherence tomography for the CSF scaffold.

**Figure 3.**
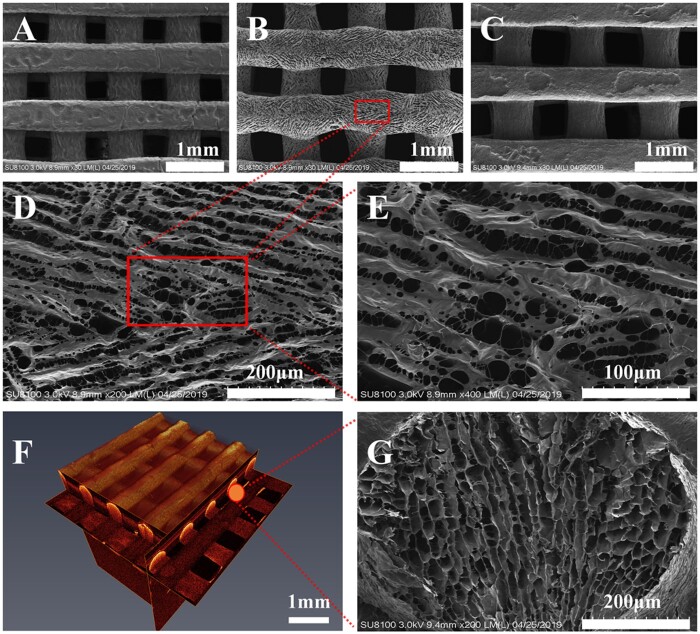
The structure of the type I collagen/silk fibroin (CSF) scaffold. **A–C**: Scanning electron microscopy of scaffold materials with different aperture sizes (due to shooting reasons, A and C showed the bottom surface of scaffold materials, because the printing bottom plate was relatively straight under contact; B showed the top surface of scaffold materials, which the small pores on the surface of scaffold materials were clearly observed); **B, D, E**: Scanning electron microscopy observations of the CSF2 (magnification: ×200, ×400 and ×800, respectively); **F**: Optical coherence tomography (observations on the shape rule of scaffold material and uniform distribution of pore diameter); **G**: Cross-section of CSF2 (×200).

Figure 2A showed the three-dimensional digital model of CSF fibroin scaffold. The real scaffold after 3D printing and freeze-drying treatment had porous network structures and good connectivity between the apertures. OCT images showed that the regular internal structure of the scaffolds (Figs. 2B and 3F).

### SEM observation results

SEM showed that the morphologies of the prepared scaffold materials were regular with CSF1, CS2, CS3. The aperture diameter of each group was 421 ± 27 μm, 579 ± 36 μm and 707 ± 43 μm, respectively. Micropores were presented and the pores connected with each other (Fig. 3A–E, G).

### Porosity and hydrophilicity

The porosity of CSF1, CSF2, CSF3 scaffolds was 69.8 ± 2.2%, 80.1 ± 2.8% and 86.5 ± 3.3%, respectively. The hydrophilicity of CSF1, CSF2, CSF3 scaffolds was 727.7 ± 28.0%, 782.7 ± 39.5% and 830.5 ± 65.2%, respectively.

### Mechanical testing results

**Figure 4.**
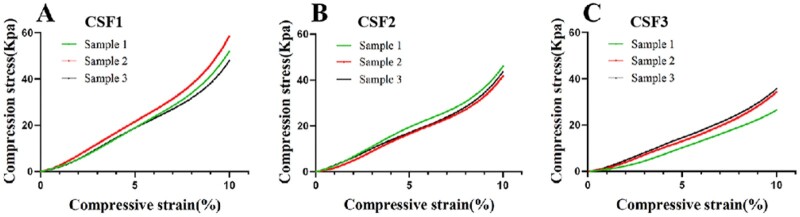
Compressive stress–strain curves of the type I collagen/silk fibroin (CSF) scaffolds. **A, B, C** represented different aperture sized CSF scaffolds. The compression stress–strain test results showed that the scaffold materials were viscoelastic. There was no significant difference among these scaffolds (within the group).

The CSF scaffolds with different aperture sizes were all flexible and elastic. They could all automatically return to their original shapes after the external forces being removed. The compression stress–strain curves of the CSF1, CSF2, CSF3 scaffolds were shown in Fig. 4. As the aperture size was increased, only very slight changes in the compression strength was observed. The averages Youngs’ elastic moduli of these three scaffolds were 546.6 ± 79.8 kPa, 431.0 ± 21.7 kPa and 356.9 ± 46.1 kPa, respectively. The aperture size of the CSF scaffolds did not affect the mechanical strength of the scaffolds.

### HDPCs–CSF scaffolds culture results

**Figure 5.**
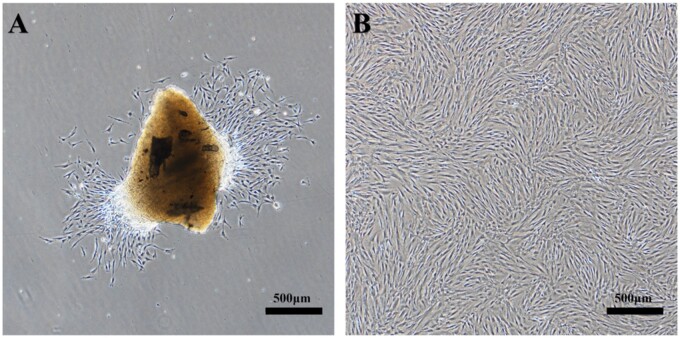
Primary HDPCs isolation and expansion *in vitro*. **A**: HDPCs migrated out of the explants onto the surface of the culture flasks after 3 days. There were some spindle-shaped or oval-shaped cells; **B**: Cells at passage 3 and ready for experiments.

HDPCs were isolated successfully from human dental pulp tissue using outgrowth/explants culture method. Most of the HDPCs were round or spindle-shaped, and the cells were arranged radially (Fig. 5A). The cells were passaged for the first time after grew to about 80–90%, and then cells were passaged every 4–5 days. The cells showed regular long fusiform morphology when passaged to the third generation (Fig. 5B).

### SEM observation results on the adhesion and proliferation of HDPCs on the scaffolds

**Figure 6.**
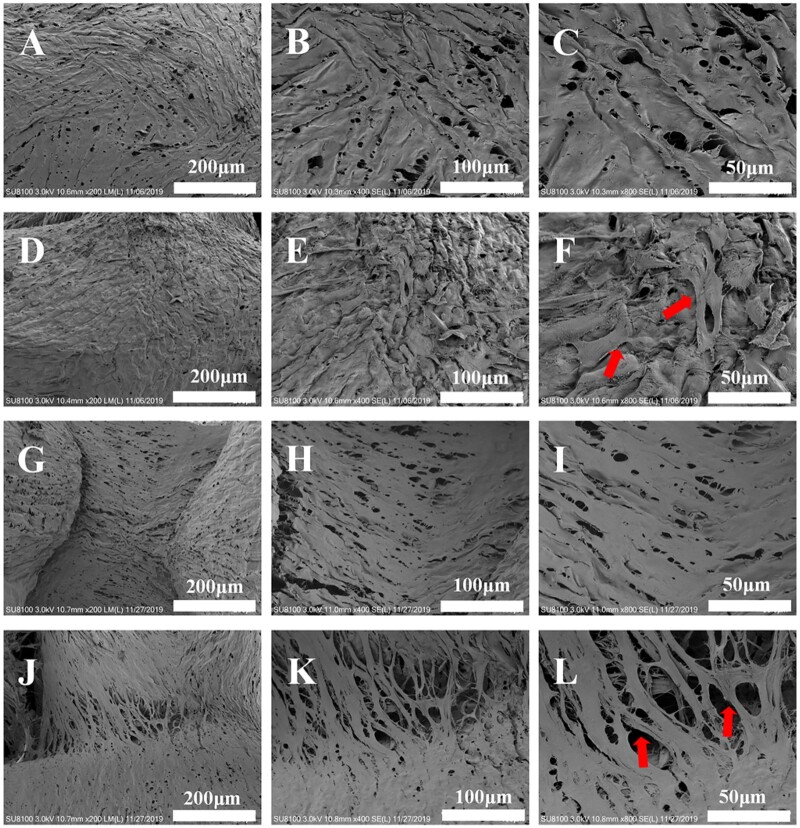
Adhesion and proliferation of HDPCs seeded with collagen/silk scaffold material (CSF2, **A–C**: simple culture medium plus scaffold material for 1 day; **D–F**: simple culture medium plus scaffold material plus HDPCs for 1 day; **G–I**: simple culture medium plus scaffold material for 7 days; **J–L**: simple culture medium plus scaffold material plus HDPCs culture for 7 days; ×200, × 400, ×800). Red arrows indicated cell distribution on the scaffolds.

SEM showed that HDPCs adhered to the surface of scaffolds, and cells protruded pseudopodia after 24 h. After 7 days, the cells grew well on the surface of scaffolds, formed multiple layers. The cells stretched sufficiently, and connected with each other. Meanwhile, HDPCs adhered to the aperture adjacent area of the scaffolds with spherical particle deposition (Fig. 6).

### Quantitative analysis of cell proliferation on the scaffolds

**Figure 7.**
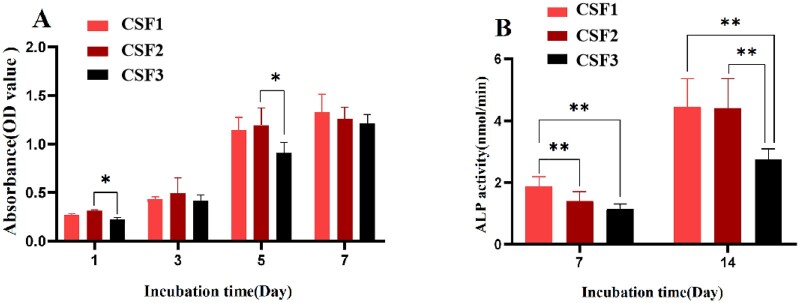
Proliferation and ALP activity of HDPCs on the collagen/silk fibroin scaffold. **A**: Effects of scaffolds with different aperture sizes on the proliferation of HDPCs after 1, 3, 5 and 7 days culture. **B**: Effects of scaffolds with different aperture sizes on the ALP activity of HDPCs. (*P < 0.05, **P < 0.01).

Figure 7A showed that the cell proliferation of each group increased with time. The value in the CSF3 group was significantly smaller than the CSF2 group after 1 day and 5 days of culture (*P *<* *0.05). After 7 days of culture, there was no significant difference among three groups (*P *>* *0.05). In three time points, the proliferation pattern of HDPCs in the three groups was similar. From 1 to 3 days, the cells proliferated slowly. Cell proliferation was significantly accelerated from the third day. At the fifth day of culture, the value of each group increased significantly compared with that on the third day, and there were significant differences between CSF2 and CSF3 groups (*P *<* *0.01). When cultured for 7 days, the proliferation rate slowed down, and there was no significant difference among these groups.

### ALP activity of HDPCs on the scaffolds

Figure 7B showed the ALP activity of HDPCs in the CSF1 group was higher than that in the CSF2 and CSF3 groups after 7 days culture (*P *<* *0.01). After 14 days culture, the ALP activity in the CSF1 group was still significantly higher than that in the CSF3 group (*P *<* *0.01), and ALP activity in the CSF2 group was also significantly higher than that in the CSF3 group (*P *<* *0.01), but there was no statistical difference between the CSF1 group and the CSF2 group (*P *>* *0.05).

### Histological observation results

**Figure 8.**
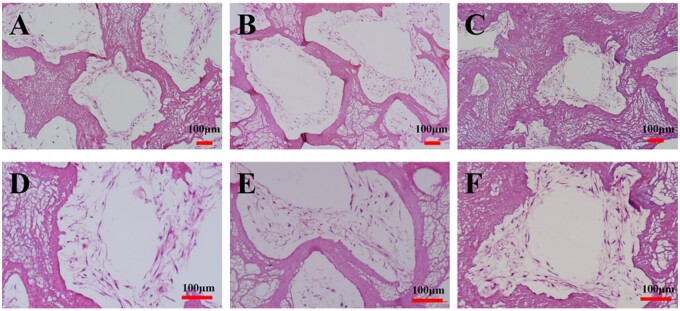
HE staining of the HDPCs–CSF scaffolds cultured for 14 days. **A, D**: CSF1 group. HDPCs attached and spreaded within and around the scaffolds aperture; **B, E**: CSF2 group. HDPCs attached and spreaded similiar as in CSF1 group, formed multilayers; **C, F**: CSF3 group. Deformed structures of the scaffolds were observed in the CSF3 group.

HE staining results showed that HDPCs grew in multilayer with large quantity and speading morphology along the scaffolds in three groups after 14 days of culture. Meanwhile, the structure of the scaffold in the CSF3 group was found to be slightly deformed during cultivation process. The scaffolds in the CSF1 and CSF2 groups showed suitable structural stability (Fig. 8).

## Discussion

Scaffolds are important tools in tissue engineering and play unique roles in regeneration and repair of damaged tissues. A variety of natural or synthetic scaffolds have been investigated in the regeneration of dentine–pulp complex [3, 7, 8, 11, 19]. As mentioned in the literature review, the scaffold prepared by 3D printing technology has the unique capability to fabricate arbitrary geometries with interconnected microchannels. It ensures the reproducible fabrication of biological tissues with minimum aberration in terms of the shape, structure and function [20, 21].

Collagen is the most abundant matrix protein in the human body. It shows excellent biocompatibility, nontoxic, low immunogenicity properties which makes it highly demanded in tissue engineering and regenerative medicine applications [22]. Silk fibroin is a natural polymer fibrin extracted from silk. As a naturally occurring protein-based biomaterial, it exhibits many promising characteristics, such as good biocompatibility, controllable biodegradability, moisture permeability, ideal mechanical properties, low cost and ease of use [23]. Recently, collagen and silk fibroin scaffolds have been widely applied in cartilage tissue engineering due to their superior comprehensive properties. Our group constructed a type II collagen/silk fibroin scaffold with suitable morphology to promote chondrocytes proliferation [14]. However, this scaffold still had some limitations, such as insufficient mechanical strength and hydrophilicity.

Dentine is comprised of about 20% organic material by weight. Approximately 90% of the organic material is primarily type I collagen (COL1). Therefore, a type I collagen/silk fibroin scaffold was fabricated and tested on dentine–pulp complex regeneration in this study. Results showed that the scaffold material had good biocompatibility. It promoted the proliferation and odontogenic differentiation of HDPCs, suggesting a potential application of these scaffolds in dentine–pulp complex regeneration.

Several studies have shown that the scaffold properties, such as porosity, hydrophilicity, mechanical properties, etc., influence the adhesion, proliferation and differentiation of seeding cells. Among these properties, the pore size or aperture size plays significant roles. Previous study verified that the pore size of >100–150 µm is desired for achieving better results in tissue growth. One study investigated the influence of pore size on the time required to bridge pores in thin 3D-printed scaffolds. Results showed that time to bridge a pore simply increased linearly with the overall pore size [24]. Tang *et al*. fabricated a porous polyetherimide (PEI) scaffold using a 3D printing system, and the pore size was set as 800 μm. They found that the 3D PEI scaffold showed an interconnected porous structure with the elastic modulus (941.33 ± 65.26 MPa) fell in the range of modulus for the native cancellous bone. Meanwhile, this scaffold has good biocompatibility since the cell proliferation and morphology on the 3D PEI scaffold showed better effects than those on the PEI slice [25]. Studies over the past decades have provided important information on the effects of scaffold pore size in bone, cartilage tissue engineering. However, there is no general agreement about the effects of different pore sized scaffolds on the proliferation and odontogenic differentiation of human pulp cells and dentine–pulp complex regeneration.

Therefore, we designed three different pore sized collagen/silk fibroin 3D printing scaffolds with pore diameters of 421 ± 27 μm, 579 ± 36 μm and 707 ± 43 μm, respectively. Except for the regular macropores, the scaffold was also covered with ‘honeycomb’ holes. After inoculation for 24 h, HDPCs adhered to the scaffold surface and the cells were fully stretched, indicating that HDPCs could adhere and grow on the CSF scaffolds. It was found that the aperture sizes of 421 ± 27 μm and 579 ± 36 μm scaffolds enhanced the adhesion of HDPCs better than the 707 ± 43 μm aperture sized scaffolds. A possible explanation for this might be that during HDPCs seeding onto the scaffolds, some HDPCs directly passed through the large pore of the scaffolds, adhered to the bottom of the 24-well plate, resulting in a decrease in the number of cells attached to the large pore diameter scaffold material itself. While small pore diameter facilitated cell contact to the material, ensured most cells adhering to the scaffolds. Consistent with the literature, our study also found that pore diameter affected the scaffold permeability. Previous study indicated that 3D printed ceramic scaffold pores should be larger than 390 µm with an upper limit of 590 µm to enhance bone formation [26]. In our study, after 7 days of cell-scaffold culture, the absorbance value of the CSF1 and CSF2 group was slightly higher than that of the CSF3 group. There was no significant difference among them, which may be related to the arrival of the plateau stage of seeded cells.

The effects of different pore sized scaffolds on the odontogenic differentiation of HDPCs were also investigated. As one of the early markers of odontogenic differentiation, alkaline phosphatase activity (ALP) was evaluated after 7 and 14 days cell-scaffold culture. After 7 days, the ALP activity in the CSF1 group was higher than that in the CSF2 and CSF3 groups, and the difference between them was statistically significant. After 14 days, the ALP activity of the CSF1 group and the CSF2 group was significantly higher than that of the CSF3 group (*P *<* *0.01). Similar effects were also observed by several other teams [27, 28]. All these findings confirmed that ALP involved in the process of mineral deposition and calcification of tissues. It played significant roles in dentine–pulp complex repair and regeneration. As a preliminary study aimed at exploring the potential of the CSF scaffolds in the future endodontic treatment, the results we presented in this study was evident. A limitation to our study was that specific odontogenic differentiation markers, such as dentine sialoprotein and dentine matrix protein, should be evaluated. Besides, the complex mechanisms by which CSF promoted the proliferation and ALP activity of the human dental pulp cells still need further investigation.

We also found that with the increase of culture time, there was deformation in the structure of the large pore diameter scaffold to some extent, indicating that the stability of scaffold with the large pore diameter was worse than that of scaffolds with small and the medium pore diameter during the long-term culture. Previous study suggested that when the porosity was smaller than a lower critical value, the porous scaffold exhibited shearing behavior with cracking along the maximum shear stress [29]. Another possible explanation for this was that the degradation of the scaffolds also counted [14].

The key properties such as porosity, hydrophilicity and compressive stress–strain of scaffold will also affect the adhesion, proliferation and differentiation of seeding cells. The experimental data are rather controversial, and there is no general agreement about the common process parameters. Studies have shown that porosity greater than 60% is beneficial to the long penetration of new bone, while excessive porosity (>70%) reduces the mechanical strength of the material [30]; the porosity in the range of 50–90% can better simulate the structure of cancellous bone to facilitate the growth of new bone. The effects of porosity, hydrophilicity and mechanical strength of the scaffolds on tissue engineering are complicated or even contradictory. However, increasing porosity of the scaffold will decrease the mechanical strength of the scaffold itself, and the increase of the compressive strength will decrease the porosity of the scaffold, which will affect the cell growth into the scaffold [31]. In our study, results also showed that the porosity and hydrophilicity of the large aperture sized scaffold were the largest, but the elastic modulus were the lowest compared with the other two groups. How to adjust these parameters to achieve material performance balance needs further exploration. This is an important issue for future research.

The ultimate goal for dentine–pulp complex regeneration is functional recovery of teeth to prolong their life. The dental pulp is a highly innervated and vascularized tissue. The aspects of angiogenesis and neural tissue formation for dentine–pulp complex regeneration are definitely very important. There are several studies successfully obtained regenerated connective pulp-like tissues with neovascularization and innervation [32, 33]. However, further understanding and improvement are still needed in developing reliable and effective approaches in endodontics. The present study demonstrated a positive effect of type I collagen/silk fibroin scaffolds on the proliferation and odontogenic differentiation of HDPCs. In future, we would like to explore the effects of this scaffold on neovascularization and innervation which are very important in dentine–pulp complex regeneration research and therapeutics. It is clear that a deep understanding of the mechanisms on the self-renewal, microenvironmental homeostasis and multi-differentiation of HDPCs would be very useful.

## Conclusions

The type I collagen/silk fibroin scaffold material prepared by low-temperature deposition 3D printing had good biocompatibility and stimulated the adhesion and proliferation of HDPCs. The scaffolds with the aperture sizes of 421 ± 27 μm and 579 ± 36 μm enhanced the adhesion and ALP activity of HDPCs better than the 707 ± 43 μm aperture sized scaffolds. This study confirmed the feasibility of the type I collagen/silk fibroin scaffolds usage in the dentine–pulp complex regeneration. Meanwhile, in spite of the limitations, the present study provided useful aperture size parameters of the scaffold for HDPCs adhesion and odontogenic differentiation.

## Funding

This work was supported by the Natural Science Foundation of Tianjin City of China (grant number 18JCYBJC27000); the National Natural Science Foundation of China (grant number 11972198); the State Key Laboratory of Medicinal Chemical Biology (grant number 2018012); the Beijing Key Laboratory of Tooth Regeneration and Function Reconstruction (grant number KFKT2017008); and the Tianjin Health and Family Planning Commission of the People’s Republic of China (grant numbers ZD20016, 2014KY24 and 2015KY23).

*Conflict of interest statement*. None declared.

## Notes

**Clinical significance:** This study developed a 3D printed type I collagen/silk fibroin (CSF) scaffold and evaluated the effects of this scaffold on the proliferation and differentiation of human dental pulp cells (HDPCs). Results indicated that CSF scaffolds were promising candidates in dentine–pulp complex regeneration.
